# Combinatorial effect of epirubicin and 5-fluorouracil in the treatment of temozolomide-resistant glioblastoma cells

**DOI:** 10.55730/1300-0152.2799

**Published:** 2026-03-13

**Authors:** Mehrsa BAYAT, Muhlis AKMAN, Türker KILIÇ, Timuçin AVŞAR

**Affiliations:** 1 Neurooncology Laboratory, Faculty of Medicine, Bahçeşehir University, İstanbul, Turkiye; 2 Department of Neurosurgery, Faculty of Medicine, Istinye University, İstanbul, Turkiye; 3 Department of Medical Biology, Faculty of Medicine, Bahçeşehir University, İstanbul, Turkiye

**Keywords:** Glioblastoma, chemoresistance, temozolomide, epirubicin, 5-fluorouracil, transcriptomics

## Abstract

**Background:**

Glioblastoma (GBM) is a highly aggressive form of brain tumor characterized by rapid proliferation and invasiveness. It is associated with a poor prognosis due to acquired resistance to temozolomide (TMZ). In this study, we investigated whether a combination of epirubicin, 5-fluorouracil (5-FU), and TMZ could improve TMZ sensitivity in resistant GBM cells and help overcome resistance.

**Materials and methods:**

TMZ resistance was established in the U87MG cell line. The MTT assay was used to measure cell viability. Reactive oxygen species (ROS) and apoptosis were measured using flow cytometry. RNA-seq was used to evaluate genomic changes based on treatment with drugs alone or in combination.

**Results:**

We demonstrated that the triple-drug combination significantly reduced cell viability. The biochemical pathways involved revealed that this combination therapy significantly increased the generation of ROS. The RNA-seq analysis indicated that combination therapy effectively suppressed cell cycle regulatory pathways, enhancing cell cycle arrest and promoting apoptosis in TMZ-resistant cells.

**Conclusion:**

These findings underscore the potential viability of integrating epirubicin and 5-FU with TMZ to improve therapeutic outcomes in patients suffering from chemoresistant GBM. These combination therapies could represent an important advance in the treatment of this challenging malignancy.

## 1. Introduction

Glioblastoma (GBM) is the most common type of primary malignant brain tumor (Ohgaki and Kleihues, 2005). Only 5.1% of patients with GBM survive for 5 years after diagnosis, indicating a very poor prognosis. The pathogenesis of GBM is characterized by uncontrolled cellular proliferation, extensive infiltration into surrounding tissue, a propensity for necrosis, enhanced angiogenesis, resistance to apoptosis, and marked genetic instability. Major obstacles to effective treatment include tumor heterogeneity, widespread invasion of adjacent healthy brain tissue, and resistance to currently available therapeutic modalities (Ostrom et al., 2018; Dymova et al., 2021).

The currently available treatment options include surgery, radiation therapy, and temozolomide (TMZ) as chemotherapy. TMZ is frequently administered alongside radiation therapy for 6 weeks during the initial phase of treatment, followed by six cycles of TMZ alone for 5 days every 28 days. Compared with patients treated with radiation alone, with a median survival of 12 months, the median survival of this standard regimen was 14.6 months (Stupp et al., 2009). Despite this aggressive trimodal approach, disease recurrence is observed in approximately 90% of patients within months of treatment initiation (Floeth et al., 2005; Stupp et al., 2009). Upon recurrence, additional surgical resection is often performed when feasible. Other therapeutic strategies include rechallenge with alkylating agents such as TMZ, platinum-based chemotherapeutics, or vascular endothelial growth factor (VEGF) inhibitors (Pavon et al., 2014; Messaoudi et al., 2015). Nevertheless, GBM remains extremely difficult to treat.

Anthracyclines are a widely used class of chemotherapeutic agents for the treatment of solid tumors, including breast and ovarian cancers (Biganzoli et al., 2002; Martín et al., 2008). Epirubicin, an anthracycline derivative, has been identified as an FDA-approved drug exhibiting pronounced cytotoxic effects on primary tumor cells isolated from surgically resected GBM samples and maintained in stem cell cultures in vitro (Jiang et al., 2014). Epirubicin is particularly active during the S phase of the cell cycle and affects tumors by interfering with DNA synthesis and function (Cersosimo and Hong, 1986). It is activated by intercalation into DNA strands to generate complexes that inhibit RNA and DNA synthesis. In addition, the generation of oxygen free radicals and the induction of DNA cleavage via topoisomerase II inhibition result in the activation of cell death pathways (Bonadonna et al., 1993).

5-Fluorouracil (5-FU) is another widely used anticancer agent that inhibits thymidylate synthase and disrupts RNA and DNA synthesis. It is most employed in the treatment of gastrointestinal malignancies (Pandey et al., 2020; Shinde et al., 2020). Uracil is essential for nucleic acid synthesis and, consequently, for tumor growth. As an analog of the naturally occurring pyrimidine uracil, 5-FU is metabolized through the same biochemical pathways. However, unmetabolized 5-FU is unable to directly bind DNA, which is the primary target site in both tumor and normal mammalian cells. The cytotoxic effects of 5-FU on nucleotide metabolism are observed only after its anabolism in actively proliferating cells (Diasio and Harris, 1989). Additionally, RNA synthesis can be inhibited through the incorporation of abnormal RNA structures (Akça and Özeş, 2002). Although 5-FU has demonstrated efficacy in the treatment of various cancers, it is not routinely included in standard therapeutic regimens for GBM.

Investigating the variables affecting resistance and response could help improve different approaches and patient outcomes. Recent studies have shown that 5-FU has the potential to be an effective therapeutic agent when delivered to brain tumors by using cutting-edge drug delivery methods, such as liposomes (Lakkadwala and Singh, 2018) or nanoparticles (Pandey et al., 2020; Shinde et al., 2020). In addition, ex vivo treatment is being developed and gene therapy for GBM has been performed (Philbrick and Adamson, 2019).

In the present study, the therapeutic effects of epirubicin and 5-FU were investigated in comparison with TMZ monotherapy. The effects of these agents on cell viability, apoptosis, and reactive oxygen species (ROS) production were evaluated in TMZ-resistant GBM cells. Additionally, RNA sequencing analysis was performed to elucidate differential gene expression profiles induced by combination treatment versus TMZ alone. These analyses were conducted to improve our understanding of the molecular mechanisms underlying the efficacy of combinatorial therapeutic strategies in GBM.

## 2. Materials and methods

### 2.1. Cell culture

The human GBM cell lines U87MG and HUVEC were grown in monolayers in 25T flasks using Dulbecco’s modified Eagle medium - high glucose (DMEM) (GIBCO Thermo Fisher Scientific, Waltham, MA, USA), supplemented with 10% fetal bovine serum (FBS) (Thermo Fisher Scientific, USA) and 1% penicillin (PS-B) (Capricorn Scientific GmbH, Ebsdorfergrund, Germany) for the U87MG cells. The cells were maintained in 5% CO_2_ at 37 °C. To study the effects of combination drugs on TMZ (MedChemExpress LLC, Monmouth Junction, NJ, USA) resistance, U87MG cells were exposed to 200 μM TMZ every 3 days for up to 16 days, after which cells were harvested at designated time points (3, 6, 9, 12, and 16 days). TMZ-resistant U87MG cells were developed through this repeated exposure protocol. Cell viability assays (MTT) were performed 24 and 48 h posttreatment; the 48-h time point was used for IC_50_ determination as it provided more consistent dose–response relationships.

### 2.2. Drug treatment

TMZ-sensitive and TMZ-resistant U87MG cells were maintained in a complete medium for 24 h to allow cell attachment; then the resistant cells were treated with TMZ alone for 24 h. Secondly, U87-TMZ-resistant cells were seeded in a 96-well plate and incubated for 24 h in DMEM. The U87-TMZ-resistant cells were treated with TMZ (MedChemExpress LLC, USA), 5-FU (MedChemExpress LLC, USA), or epirubicin (MedChemExpress LLC, USA) alone, or a combination of TMZ + 5-FU, TMZ + epirubicin, or TMZ + epirubicin + 5-FU. The concentration of 5-FU and epirubicin utilized was not greater than the IC_50_ value. Treated cells were incubated for 24 and 48 h before being subjected to various analyses. All drugs were dissolved in sterile dimethyl sulfoxide (Genaxxon bioscience GmbH, Ulm, Germany) at a final concentration of less than 0.1% and stored at −20 °C for future use.

### 2.3. MTT cellular proliferation assay

Cell viability and proliferation were assessed using an MTT assay. U87MG and U87-TMZ resistant cells were seeded in a 96-well plate and incubated for 24 h in DMEM/F12 (Gibco Thermo Fisher Scientific, USA) medium to ensure cell attachment. U87MG cells were treated with TMZ, while U87-TMZ-resistant cells received treatments of TMZ, 5-FU, or epirubicin alone, or combinations of TMZ + 5-FU, TMZ + epirubicin, or TMZ + epirubicin + 5-FU. Additionally, HUVEC cells were treated with mixed drugs. For treatment with TMZ and 5-FU alone, cells were exposed to various concentrations for TMZ (1 nM to 1000 μM) and for epirubicin (0.1 μM to 100 μM). The combination therapies employed 200 μM TMZ along with concentrations of 5-FU of 0.5 μM and 2 μM and epirubicin of 0.5 μM, which are below their respective IC_50_ values. Treated cells were incubated for 24 and 48 h. At the end of this incubation period, 10 μL of MTT solution (M6494, Thermo Fisher Scientific, USA) prepared as 5 mg/mL in phosphate-buffered saline (PBS) was added to each well and further incubated for 2 h. A solubilization buffer of 0.1 M HCl and 10% SDS was added to dissolve the formazan crystals, followed by 15 min of incubation at 37 °C. Absorbance was measured at 570 nm.

### 2.4. Reactive oxygen species

Baseline intracellular ROS levels were measured using the Cellular Reactive Oxygen Species Detection Assay Kit based on 2′,7′-dichlorodihydrofluorescein diacetate (DCFH-DA). The assay was performed according to the manufacturer’s instructions. Cells were seeded in six-well plates overnight before the experiments and treated with drugs in triplicate, incubating for 24 h at 37 °C in a 5% CO_2_ incubator. The next day, cells were harvested at the designated time point. The medium was removed and the cells were washed once with PBS. To determine intracellular ROS levels after treatment, 1 mL of DCFH-DA working solution was added to each well in the dark and incubated at 37 °C for 30 min. The DCFH-DA working solution was then removed and the cells were trypsinized. The mixture was centrifuged at 1000 rpm for 10 min. The cells were washed once with DMEM and twice with 1X PBS. Cells were resuspended in 300 μL of 1X PBS and transferred to a FACS tube on ice in a dark environment. An ACEA Novocyte 3005 (Agilent) flow cytometry instrument was used to detect and analyze the results at 535 nm. For analysis of the results obtained from the flow cytometry, NovoExpress software (version 1.5, Agilent) was used.

### 2.5. Annexin V/propidium iodide assay

Cells were seeded in six-well plates, treated in triplicate, and incubated for 24 h at 37 °C in a 5% CO_2_ incubator. The medium was removed and the cells were washed with PBS before 1 mL of trypsin was added. After detachment, cells were collected. The mixture was then centrifuged at 3500 × *g* for 5 min. Following the removal of the supernatant, the cell pellet was washed twice in PBS + 2% FBS and then centrifuged twice at 3500 × *g* for 5 min. Cells were resuspended in 100 μL of 1x Annexin V binding buffer, followed by the addition of 2.5 μL Annexin V from the Apoptosis Detection Kit (Biolegend #640932) and 5 μL propidium iodide (PI). The cells were then incubated for 15 min in the dark. Finally, 400 μL of binding buffer was added to each tube. The ratio of early apoptotic, late apoptotic, and dead cells was measured by flow cytometry. Apoptosis was quantified by annexin V/PI flow cytometry using a standardized gating strategy applied to all samples (Figure S1). Cells were first gated by FSC/SSC to exclude debris, followed by FSC-A/FSC-H to remove doublets. Apoptotic and necrotic populations were then identified by quadrant analysis of annexin V versus PI staining, classifying cells as viable (annexin V^−^/PI^−^), early apoptotic (annexin V^+^/PI^−^), late apoptotic (annexin V^+^/PI^+^), or necrotic (annexin V^−^/PI^+^). Epirubicin-containing treatments resulted in a marked increase in annexin V-positive cells, with minimal annexin V^−^/PI^+^ populations indicating that cell death occurred predominantly through apoptosis rather than primary necrosis. All experiments were performed in biological triplicates (n = 3) and representative flow cytometry plots are shown in Figure S1.

### 2.6. Colony formation assays

The effects of combinatorial drug treatments on cancer-initiating stem cells were assessed using colony formation assays. Cells were seeded in six-well plates at 500 cells/well density. The next day, the cells were treated in triplicate with temozolomide, epirubicin, temozolomide + 5-FU, temozolomide + epirubicin, or temozolomide + epirubicin + 5-FU at designated dosages. After 10 to 14 days, the cultured cells underwent two washings with 1X PBS and were subsequently fixed using ice-cold methanol for 5 min. Next, 1% aqueous crystal violet solution (Sigma-Aldrich, #V5265-) was diluted with 1X PBS to 0.25% and added to the cells. The cells were incubated for 30 min at room temperature. The crystal violet was removed and the cells were washed under tap water, dried, and counted.

### 2.7. Sample preparation for RNA sequencing

Total RNA was extracted from U87-R cells (5 × 10^5^ cells per replicate) using TRIzol reagent (Zymo Research, Irvine, CA, USA) according to the manufacturer’s protocol. RNA extraction was performed on biological triplicates for each experimental group (n = 3 for U87-S; n = 3 for U87-R). The quality, purity, and structural integrity of isolated RNA were assessed using fluorometric quantification and capillary electrophoresis. RNA concentration was measured using a Qubit 3 Fluorometer (Thermo Fisher Scientific) and RNA integrity was evaluated using an Agilent 2100 Bioanalyzer. Samples with an RNA integrity number (RIN) ≥ 8.0 were used for library preparation.

### 2.8. Library preparation and sequencing

The library preparation for the isolated RNA materials was conducted utilizing the Illumina Stranded mRNA Prep Kit, a sophisticated tool designed for optimal mRNA profiling. The methodology starts with the selective capture of polyadenylated (polyA) mRNAs from total RNA, ensuring that only mature, full-length mRNAs are included in the library. Following capture, the mRNAs undergo random fragmentation, breaking the mRNA into smaller, manageable pieces that facilitate subsequent amplification and cDNA synthesis. After fragmentation, the mRNA is purified to remove any residual contaminants, ensuring that the integrity and quality of the mRNA are maintained throughout the preparation process. The purified fragmented mRNA is then reverse transcribed to synthesize complementary DNA (cDNA), first involving the enzyme reverse transcriptase synthesizing the primary cDNA chain from the mRNA template, followed by the creation of a second strand to form a double-stranded cDNA (dsDNA) library, which is ready for amplification and sequencing. These processes collectively enable the generation of high-quality cDNA libraries that are fundamental for downstream applications in gene expression analysis, RNA sequencing, and other genomic investigations.

### 2.9. Bioinformatics analysis

Raw sequencing reads were assessed for quality using FastQC. Quality metrics including data volume, read quality scores, GC content distribution, k-mer distribution, and potential adapter contamination were evaluated for each sample. Low-quality bases and adapter sequences were trimmed using Trimmomatic (v0.39) with quality-based parameters to minimize bias in downstream analyses (Bolger et al., 2014). Trimmed reads were aligned to the human reference genome (GRCh38) using the Illumina DRAGEN Bio-IT platform with the RNA Pipeline workflow, incorporating splice-aware alignment. Transcript-level read counts were computed and normalized to transcript length to generate reads per kilobase (RPK) values, which were subsequently normalized to total read counts to derive transcripts per million (TPM) values. Differential gene expression analysis was performed using the edgeR package in R with a quasilikelihood model to identify differentially expressed genes (DEGs) (Robinson et al., 2010). Gene ontology (GO) enrichment analysis was conducted using the topGO package with Kolmogorov–Smirnov statistical tests; pathway enrichment analysis was performed using the clusterProfiler package^1^(Yu et al., 2012). Principal component analysis (PCA) was conducted to assess sample clustering and confirm reproducibility among biological replicates. All statistical analyses and data visualizations were implemented using custom R scripts.

### 2.10. Statistical analysis

IC_50_ values were determined using nonlinear regression analysis. Colony formation was quantified using one-way ANOVA followed by multiple comparison tests. The data are presented as mean ± standard deviation (SD). Statistical significance was defined as *p < 0.05 and ****p < 0.0001. For RNA-seq analysis, differentially expressed genes were identified using thresholds of |log_2_ fold change| > 1 and false discovery rate (FDR)-adjusted q-value < 0.05.

## 3. Results

### 3.1. U87MG cells acquired resistance to temozolomide over time

**Figure 1:**
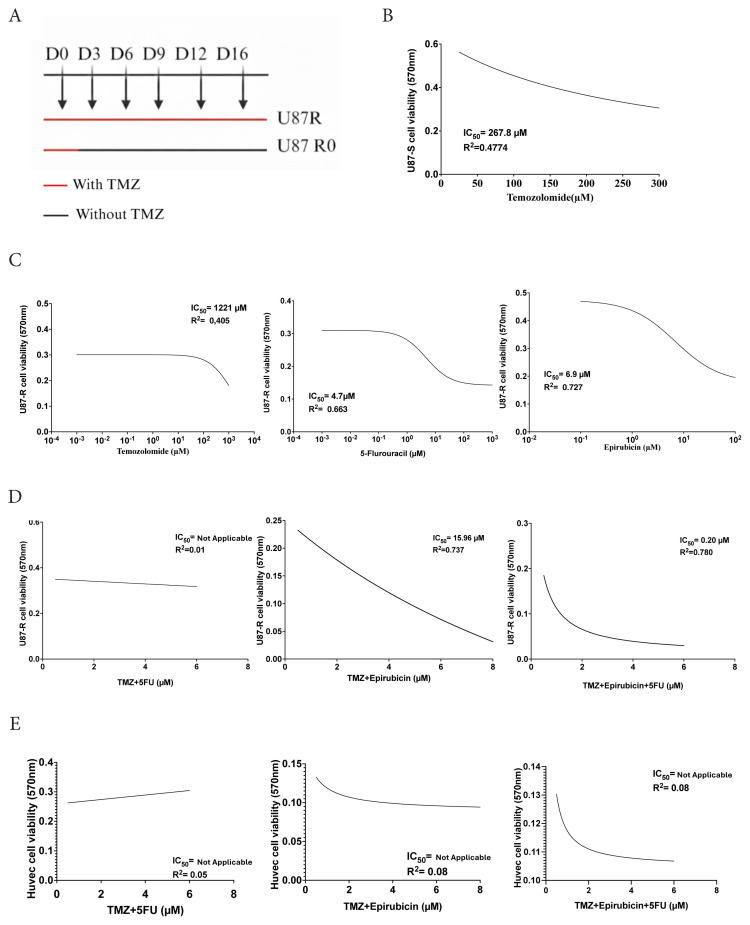
Generation of TMZ-resistant U87 cells and the IC_50_ values of the treatments are shown. A) Experimental design of TMZ treatment to obtain TMZ-resistant cells, cells treated for 16 days with 200 μM respectively. B, C) Cell viability determined using MTT. D) Combinatorial effect of TMZ, 5-FU, and epirubicin on cell viability in U87MG-TMZ-resistant cells. E) Combinatorial effect of TMZ, 5-FU, and epirubicin on cell viability in HUVEC cells.

U87MG GBM cells were treated with 200 μM TMZ every 3 days for up to 16 days (Figure 1A). To quantitatively validate the establishment of TMZ resistance, IC_50_ values were determined for both parental and resistant cell populations. U87-S (parental) cells exhibited an IC_50_ of 267 μM (Figure 1B), whereas U87-R cells displayed an IC_50_ of 1221 μM (Figure 1C), representing a 4.6-fold increase in TMZ resistance. This substantial shift in IC_50_ confirms the successful establishment of a TMZ-resistant phenotype following the 16-day exposure protocol.

To evaluate the effects of 5-FU and epirubicin on U87-R cells, we performed MTT cell viability assays with different concentrations of 5-FU (1 nM to 1 mM) and epirubicin (0.1 μM to 100 μM) both 24 and 48 h posttreatment. The 48-h data were used for subsequent analyses as they yielded more consistent dose–response curves for IC_50_ determination. Nonlinear regression analysis was performed (Figure 1C). Based on these results, 200 μM TMZ and IC_50_ concentrations of 5-FU and epirubicin were selected for combination studies.

We then evaluated cytotoxicity induced by combined administration of TMZ + 5-FU, TMZ + epirubicin, and TMZ + epirubicin + 5-FU after 48 h. As shown in Figure 1D, nonlinear regression analysis revealed significant differences between treatments. The combinations of TMZ + epirubicin and TMZ + epirubicin + 5-FU demonstrated significantly enhanced antitumor effects compared to TMZ alone. Notably, 5-FU alone did not significantly increase TMZ efficacy in U87-R cells. Additionally, cytotoxicity was evaluated in HUVEC cells to assess potential off-target toxicity. As shown in Figure 1E, no significant cytotoxicity was observed in HUVEC cells, indicating that the combined treatments were not toxic to nontumor endothelial cells.

### 3.2. Triple combination therapy enhances apoptosis and ROS in resistant cells

**Figure 2:**
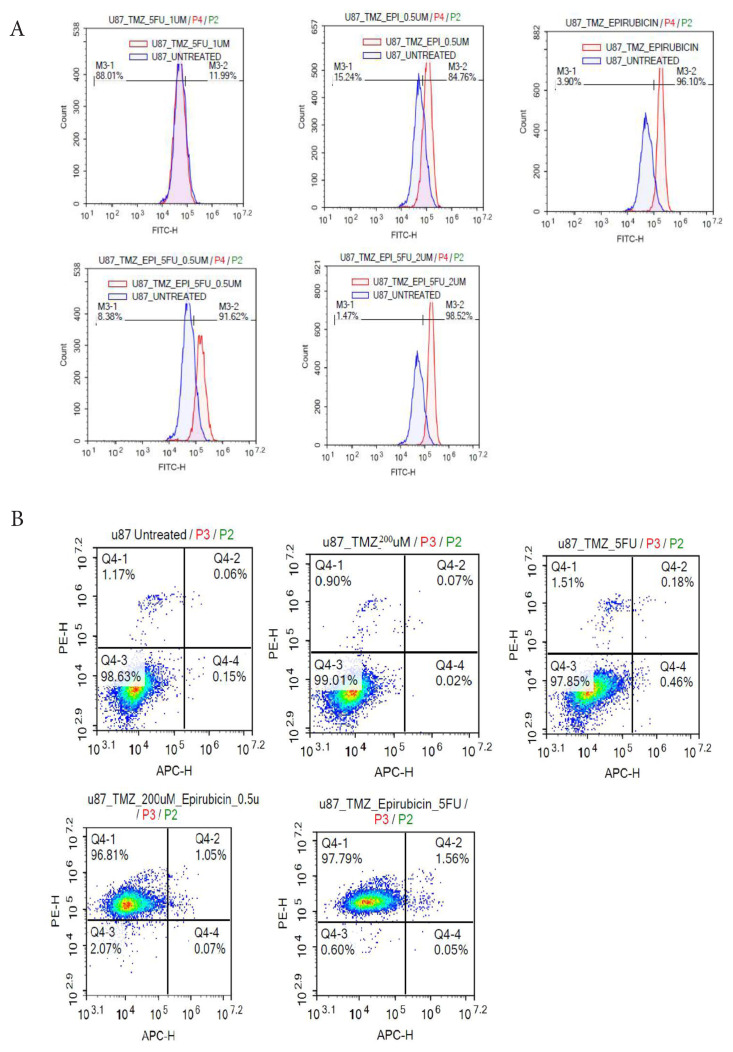
Effects of TMZ, epirubicin, and 5-FU on ROS generation in U87-TMZ-resistant cells. B) Dot plots of Annexin V and PI staining showing flow cytometric analysis.

A potential mechanism through which drug treatments induce apoptosis is illustrated in Figure 2. The production of ROS was assessed after treating U87-R cells with various agents, including TMZ, epirubicin, TMZ + 5-FU, and TMZ + epirubicin + 5-FU. ROS levels were compared to those in untreated control cells. Figure 2A presents the quantification of ROS in treated U87-R cells relative to the controls. Notably, treatments with TMZ alone and TMZ + 5-FU did not result in significant ROS induction. In contrast, epirubicin alone and the combination of TMZ + epirubicin + 5-FU demonstrated the highest ROS production, consistent with the known mechanism of anthracyclines, which induce oxidative stress through redox cycling and mitochondrial dysfunction (Sun et al., 2022; Yang et al., 2024).

Annexin V/PI staining assays were performed to confirm apoptosis induction. U87-R cells were treated with TMZ alone, epirubicin alone, and various combinations for 24 h, followed by flow cytometric analysis using annexin V and propidium iodide (PI) staining. As depicted in Figure 2B, untreated cells and those treated with TMZ alone or TMZ + 5-FU did not exhibit significant apoptosis. However, cells treated with epirubicin alone and the combination of TMZ + epirubicin + 5-FU displayed substantial apoptotic activity, with dead cell populations of 98.92% and 97.79%, respectively. These findings indicate that elevated ROS production correlates with increased apoptosis, supporting oxidative stress as a key mechanism underlying the cytotoxic effects of epirubicin-containing regimens.

Notably, while U87-R cells exhibited an IC_50_ of 6.9 μM for epirubicin monotherapy, combination treatment with 200 μM TMZ dramatically potentiated epirubicin’s cytotoxic effects, achieving apoptosis rates exceeding 97% with only 0.2 μM epirubicin—a concentration representing approximately 3% of the single-agent IC_50_. This striking enhancement of cell death at markedly sub-IC_50_ epirubicin concentrations suggests a favorable drug interaction, potentially exceeding simple additive effects, although formal synergy analysis using established methods such as the Chou–Talalay combination index was not performed in the present study. Such synergy suggests that TMZ sensitizes resistant cells to epirubicin-induced oxidative damage, possibly by disrupting compensatory survival or antioxidant defense pathways, thereby restoring drug sensitivity at otherwise ineffective concentrations. From a translational perspective, combination strategies enabling substantial dose reductions while maintaining therapeutic efficacy could minimize systemic toxicity and improve patient tolerability in GBM treatment.

### 3.3. Combination of TMZ, epirubicin, and 5-FU significantly reduces colony formation of GBM cells

**Figure 3:**
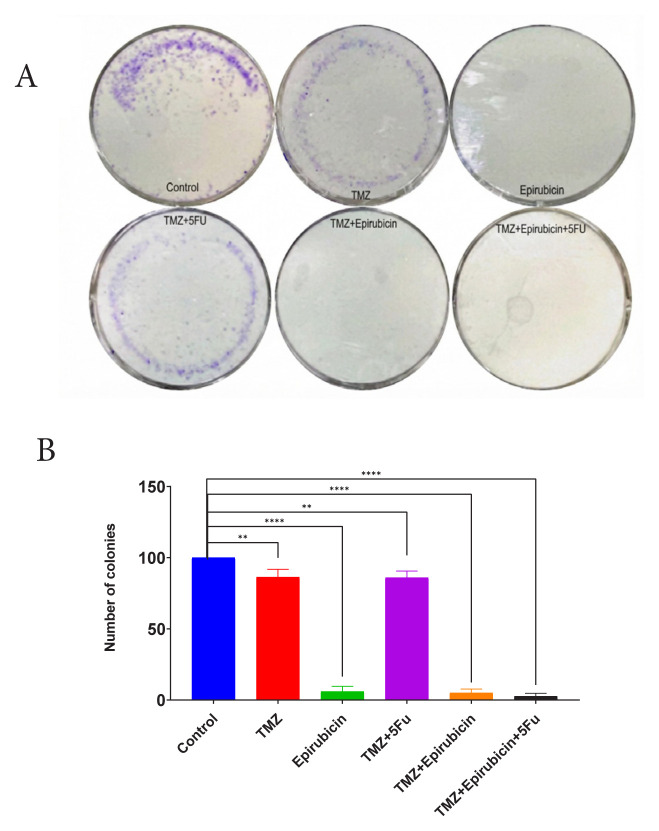
Clonogenic assay results show that combination therapy is highly effective against colony formation. A) Cells were treated with drugs for 10–14 days. Survival clones were determined under a microscope. Pictures are representative of one of three experiments. B) Data were analyzed and represented the mean ± standard deviation; *p < 0.05, ****p < 0.0001.

To evaluate the longer-term effects of combined therapy on the proliferative capacity of GBM cells, we assessed colony formation capability 10 days posttreatment using a clonogenic assay (Figure 3). U87-R cells were treated with TMZ, epirubicin, TMZ + 5-FU, TMZ + epirubicin, or TMZ + epirubicin + 5-FU and subsequently cultured in normal medium for 10 days. Colonies were visualized by crystal violet staining (Figure 3A), and quantitative analysis was performed by measuring staining intensity following dissolution in acetic acid (Figure 3B). Relative colony formation was calculated compared to untreated controls. Treatment with TMZ alone or TMZ + 5-FU resulted in colony formation comparable to that of untreated cells. Epirubicin monotherapy produced partial inhibition with residual colony formation. Notably, the combinations of TMZ + epirubicin and TMZ + epirubicin + 5-FU markedly suppressed colony-forming ability compared to single-agent treatments. These findings demonstrate that combined therapy enhances the antiproliferative effects against U87-R cells, suggesting potential therapeutic benefit of multidrug regimens in overcoming TMZ resistance.

### 3.4. Drug combinations altered the gene expression profile of resistant GBM cells

The combination of TMZ, epirubicin, and 5-FU significantly increased DEGs. An average of 61,709,904 clean reads were generated with a quality score of 91% Q30, leading to the assembly of 12,225,126 genes. DEGs between samples were evaluated using a false discovery rate (FDR)-corrected probability of q < 0.05. Various transcript gene numbers were identified in each group based on DEGs. A heat map illustrating the top 50 upregulated and downregulated DEGs was generated, with samples and genes clustered along both axes according to their profile similarities, visualized through dendrograms. A volcano plot of all DEGs was constructed using the ggplot2 package.

**Figure 4:**
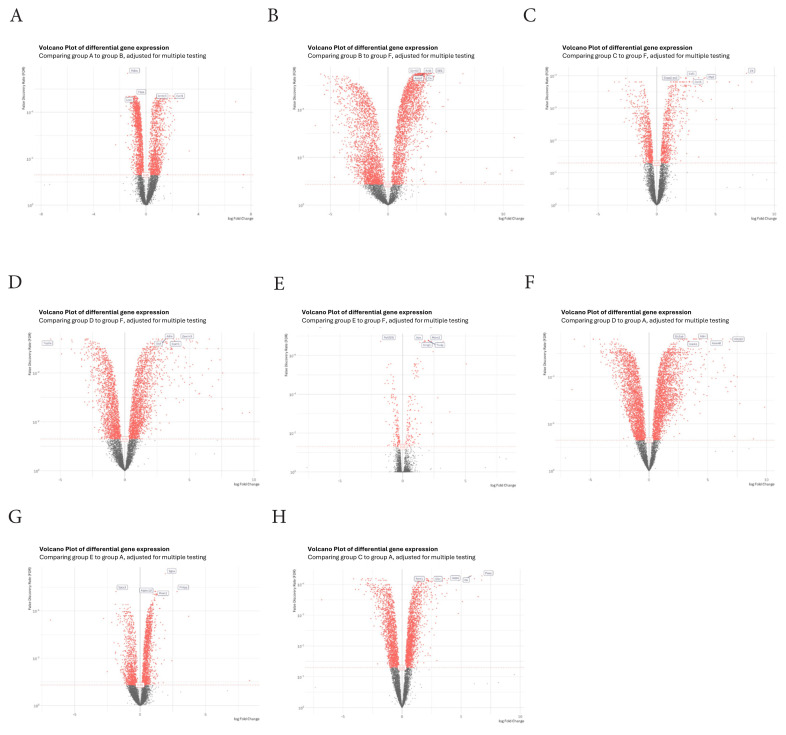
Volcano plot of all significant DEGs. Each dot on the graph represents a gene. If present, dots in red indicate genes with statistically significant (FDR < 0.05) differences between the two groups. The default set of thresholds was fold change ≥ Pathw2, p ≤ 0.05. DEGs: differentially expressed genes. a) TMZ vs. control, b) epirubicin vs. control, c) TMZ + 5FU vs. control, d) TMZ + epirubicin vs. control, e) TMZ + epirubicin + 5FU vs. control, f) TME + 5FU vs. TMZ, g) TMZ + epirubicin vs. TMZ, h) TMZ + epirubicin + 5FU vs. TMZ.

To investigate stable transcriptome changes in our resistant model, we focused on the upregulated and downregulated genes between control and TMZ-resistant models (Table S). Under 200 μM TMZ treatment, more than 8200 DEGs were identified, comprising 1253 upregulated and 1649 downregulated genes (Figure 4A). With epirubicin treatment, the expression levels of 4965 genes were altered relative to the control, with 2535 genes increased and 2430 genes decreased (Figure 4B). The combination group (TMZ + 5-FU) exhibited over 8000 DEGs compared to the control group, including 141 upregulated and 168 downregulated genes (Figure 4C). The TMZ + epirubicin group revealed 8412 DEGs compared to the control group, with 864 upregulated and 837 downregulated genes (Figure 4D). In the TMZ + epirubicin + 5-FU group, the number of upregulated and downregulated genes was 2095 and 1825, respectively (Figure 4E). Furthermore, in the TMZ + 5-FU treatment group, the expression levels of 2397 genes were altered compared to the TMZ treatment, with 1397 upregulated and 1000 downregulated genes (Figure 4F). In the TMZ + epirubicin group, 1939 genes increased and 1718 genes decreased compared to the TMZ group (Figure 4G). Under triple treatment, 8412 DEGs were identified between the TMZ + epirubicin + 5-FU and TMZ groups, with 2655 genes upregulated and 2543 genes downregulated (Figure 4H).

**Figure 5:**
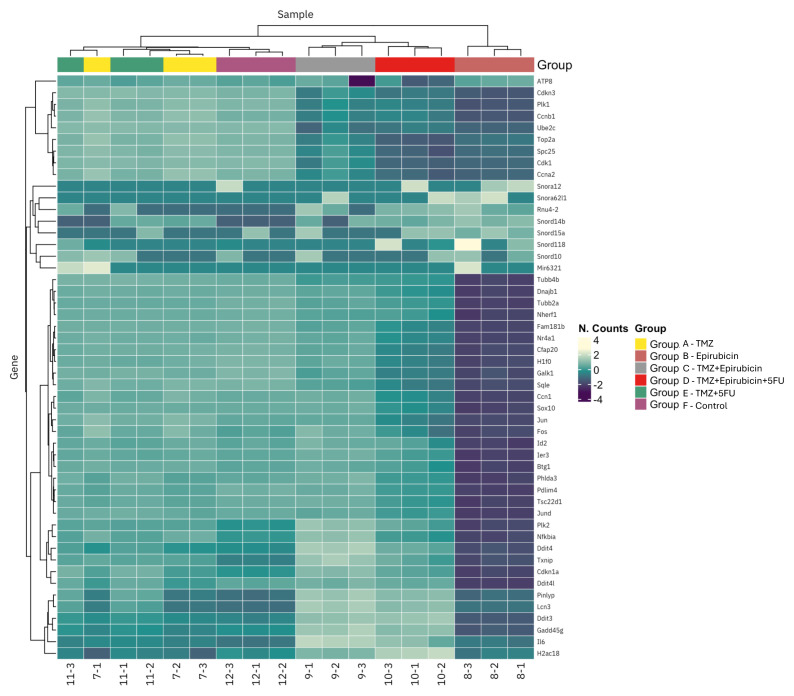
Heatmap expression profile of the top 50 genes showing the most variation between samples among RNA-seq samples. Groups are as follows: group A is temozolomide treatment, group B is epirubicin treatment, group C is temozolomide + epirubicin, group D is temozolomide + epirubicin + 5-FU, group E is temozolomide + 5-FU, and group F is the control.

As demonstrated in Figure 5, the expression profiles of the top 50 genes displaying the highest levels of variation among the samples are shown. The samples and genes in the heat map are clustered on both axes according to their profile similarities, and the similarities are visualized using dendrograms. It is important to note that only protein-coding genes are included in the heat map. The triple combination therapy (group D: temozolomide + epirubicin + 5-FU) resulted in significant upregulation of several genes including tubulin beta family members (Tubb4b, Tubb2a), the molecular chaperone Dnajb1, and the transcription factor Nfkb1 compared to the untreated controls (group F). Conversely, multiple cell cycle regulatory genes were markedly downregulated in the triple combination group, including key mitotic regulators Plk1, Ccnb1, Cdk1, and Ccna2, as well as the DNA replication and repair genes Top2a and Ube2c (Figure 5).

### 3.5. Combinatorial treatment downregulated to cell cycle-related pathways

GO functional enrichment analysis was used to determine the potential molecular mechanisms used by these 15 genes. The top 15 biological processes (BPs), molecular functions (MFs), and cellular components (CCs) are shown in Figure S2. Our analysis for TMZ + epirubicin + 5FU vs. the controls revealed that DEGs were mainly enriched in response to BPs’ and MFs’ chemical and aminoacyl-tRNA ligase activity, respectively. For CCs, DEGs were primarily enriched in the extracellular space, extracellular region, and amino acid transport complex (Figure S2A). In addition, for TMZ + epirubicin + 5FU vs. TMZ, the significantly enriched MF terms were structural molecule activity, a structural component of the ribosome, and protein binding. The predominantly enriched CC terms were cytosolic ribosome, cytosolic large ribosomal subunit, and side of membrane (Figure S2B).

**Figure 6:**
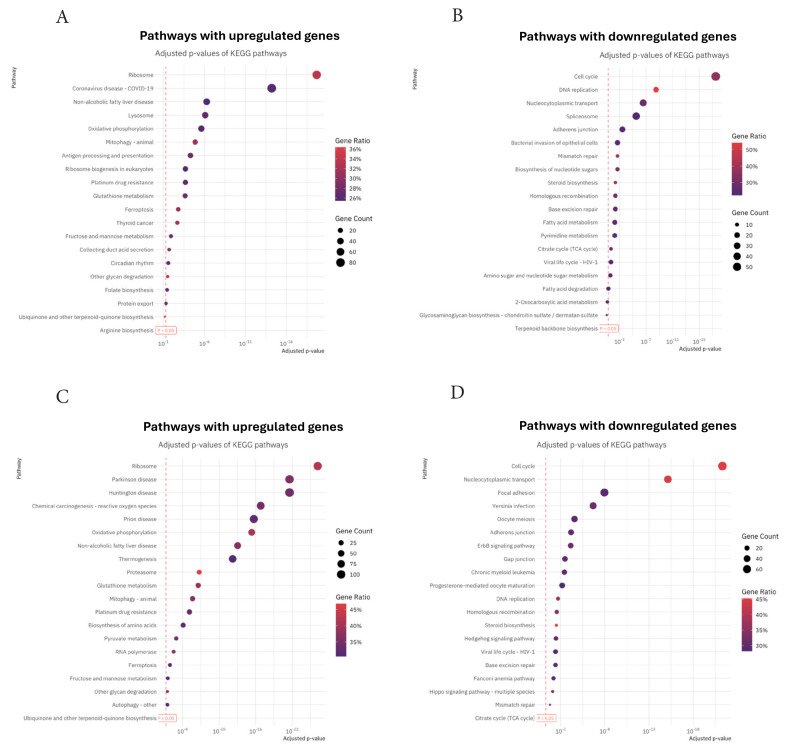
Pathway analysis of differentially expressed genes (DEGs) using the Kyoto Encyclopedia of Genes and Genomes database. (A) DEGs in the TMZ + epirubicin + 5FU group compared to the control group were associated with 78 upregulated and 32 downregulated pathways. Key upregulated pathways included ribosome, coronavirus disease-COVID-19, and Epstein–Barr virus infection. (B) Clustering of pathway groups with significant expression variability (FDR < 0.05) in the TMZ + epirubicin + 5FU vs. control comparison. The top three upregulated pathways were ribosome, Parkinson’s disease, and Huntington’s disease, while the top three downregulated pathways were cell cycle, nucleocytoplasmic transport, and focal adhesion.

Pathway analysis allows us to test whether the expression of genes encoding proteins involved in a molecular pathway shows statistically significant differences between experimental and control groups. Thus, the causes of phenotypic differences between biological groups can be investigated by examining changes in gene expression in a pathway. For pathway analysis of significantly DEGs, the Kyoto Encyclopedia of Genes and Genomes (KEGG) database was used. For the TMZ + epirubicin + 5FU vs. control group, these DEGs distributed 78 upregulated and 32 downregulated pathways, which overexpressed ribosome, coronavirus disease-COVID 19, and Epstein–Barr virus infection pathways (Figure 6A). Furthermore, within the scope of the comparison between the TMZ + epirubicin + 5FU and control groups, the pathway groups in which genes showed significant variability in expression analysis (FDR < 0.05) are clustered. Three major upregulated pathways are ribosome, Parkinson’s disease, and Huntington’s disease. Further, the top three enriched downregulated pathways are the cell cycle, nucleocytoplasmic transport, and focal adhesion (Figure 6B).

## 4. Discussion

GBM is the most common and aggressive malignant brain tumor in adults (Geraldo et al., 2019), with a median survival of approximately 16 months (Stupp et al., 2005). The development of drug-resistant cell populations in cancer is due to several factors, such as drug efflux mechanisms, metabolic changes, mutations in signaling pathways related to cell death and senescence, and epigenetic modifications (Cisternino et al., 2004). Treatment resistance is also thought to be affected by cancer stem cells, a specific group of cells capable of forming new tumors.

The term multidrug resistance (MDR) describes a cell’s ability to resist one chemotherapy drug while still being sensitive to multiple other drugs with different structures and mechanisms of action (Wu et al., 2014). MDR is a common occurrence in GBM and other cancers. While many drug-resistant phenotypes require continuous pharmaceutical treatment, drug-resistant subpopulations can emerge again when patients discontinue therapy. The gold standard treatment for GBM and astrocytoma is TMZ, a first-line alkylating agent. However, cancer cells often upregulate MDR pathways, leading to most patients not responding to TMZ during treatment (Tomar et al., 2021). The blood–brain barrier presents a significant challenge by limiting the delivery of therapeutic drugs to tumor sites. Over 90% of GBM patients experience tumor recurrence, and treatment resistance is common, which highlights the urgent need for innovative approaches and therapeutic targets in GBM treatment (Louis et al., 2021).

Most research has focused on finding novel treatments for GBM due to its aggressive nature and diversity, which make complete surgical removal nearly impossible. One approach attempted to enhance TMZ therapy by inhibiting the DNA repair enzyme O6-methylguanine-DNA methyltransferase (MGMT) using O-6-benzylguanine. However, because of their toxicity and lack of survival benefits, clinical trials were discontinued. As a result, for 20 years, the standard treatment for GBM—comprising surgery, radiation, and TMZ—remained unchanged (Stupp et al., 2005, 2017). In the present study, we successfully created a TMZ-resistant GBM cell line using a low dose of the drug over a short period. This demonstrates that resistance can develop quickly, even under relatively mild selective pressure. These findings are consistent with previous research indicating that GBM patients who have not undergone TMZ treatment before chemotherapy may develop resistance to TMZ. This resistance primarily occurs through the repair of TMZ-induced DNA lesions facilitated by MGMT (Yi et al., 2019). The observation that resistance developed in our study within only 16 days at a concentration of 200 μM indicates that the acquisition of resistance is common and occurs more easily than previously thought. This finding highlights the significant challenge of maintaining the long-term effectiveness of TMZ in treating GBM.

Numerous strategies have been investigated to overcome TMZ resistance in GBM. These strategies include targeting MGMT, blocking homologous recombination, inhibiting UBE2S, activating JNK signaling, overexpressing miR-124, and utilizing combination therapies like fisetin or TMZ–selenium conjugates (Yi et al., 2019; Ferah et al., 2023; Müller et al., 2024; McCord et al., 2025; Roy et al., 2025; Sharma et al., 2025; Wei et al., 2025; Xu et al., 2025). The goal of these approaches is to enhance the sensitivity of GBM cells to TMZ by targeting key resistance mechanisms. In the present study, we used a combinatorial drug strategy, combining TMZ with 5-FU and epirubicin. Our results showed that this combination therapy significantly reduced the IC_50_ of each drug in TMZ-resistant GBM cell lines, indicating a combination benefit. This suggests that drug combinations that target multiple pathways may effectively resensitize resistant cells to chemotherapy. The observed reduction in IC_50_ values further highlights the potential of combinatorial drug regimens to enhance treatment efficacy.

Epirubicin is an antibiotic derived from anthracycline that exhibits its activity by inhibiting replication by triggering DNA cleavage by topoisomerase II, resulting in irreversible DNA damage (Roskoski, 2024). Epirubicin is an anthracycline antibiotic that is currently approved by the FDA exclusively for the treatment of breast cancer. Nonetheless, it has been utilized off label to manage various malignancies, including non-Hodgkin’s lymphoma, Hodgkin’s lymphoma, ovarian cancer, thyroid cancer, sarcomas, and leukemias in multiple international contexts (Bayles et al., 2023). Numerous studies have demonstrated the antitumor activity of epirubicin against gliomas. In our investigation, we also evaluated the anticancer efficacy of epirubicin on GBM cell lines. High concentrations of epirubicin were ineffective in eradicating the TMZ-resistant U87 cell line, but a notable anticancer effect was achieved when combining TMZ with epirubicin at lower doses. A randomized phase 3 trial indicated that the combination of cyclophosphamide, 5-FU, and epirubicin is as effective as cyclophosphamide, 5-FU, and doxorubicin. However, the former regimen demonstrated improved tolerability among patients with advanced breast cancer (Chauvergne et al., 1988). Our findings from the combination therapy are consistent with the previously reported results.

We conducted an RNA-seq experiment on U87-TMZ-resistant cells and 5-FU and epirubicin drug-induced TMZ-resistant cells. Following a comprehensive analysis of the genes that were consistently significantly upregulated and downregulated across all experimental groups, we identified 15 commonly altered genes. A comparative analysis of control cells versus the TMZ treatment group about other cohorts indicated that the ribosomal biogenesis pathway exhibited the most pronounced alterations associated with gene upregulation. In contrast, the cell cycle regulatory pathway was predominantly impacted by gene downregulation. The action of TMZ can be classified as either p53-dependent or p53-independent, indicating that it may induce G2/M cell cycle arrest via p53 pathways or through mechanisms of mitotic catastrophe that are devoid of p53 involvement (Roos et al., 2007). Research investigating the synergistic effects of various pharmacological agents in conjunction with TMZ for treating GBM has demonstrated that combination therapy enhances cell cycle arrest during the G2/M phase, leading to increased apoptosis in cancer cells (Yi et al., 2024; Pawlak and Majchrzak-Celińska, 2025). RNA sequencing data provide compelling evidence that combination therapy markedly suppresses the activity of cell cycle-associated pathways, indicating that enhanced cell cycle arrest is a key contributor to the eradication of TMZ-resistant cancer cells. This transcriptomic modulation is further supported by flow cytometry analyses, which reveal significantly elevated apoptosis rates following combination treatment. Importantly, while TMZ alone or in combination with 5-FU failed to induce substantial oxidative stress, treatments containing epirubicin—either alone or in triple combination with TMZ and 5-FU—elicited pronounced ROS production. This increase in oxidative stress is consistent with the established mechanism of anthracyclines, which promote ROS generation through redox cycling and mitochondrial dysfunction. Collectively, these findings suggest that the therapeutic efficacy of the combination regimen arises from the convergence of cell cycle inhibition, apoptosis induction, and anthracycline-driven oxidative stress (Sun et al., 2022; Yang et al., 2024).

Recent studies showed that targeting genes and their protein expressions can overcome TMZ resistance and radio resistance. c-Fos, although a poor predictor for several cancers such as squamous cell lung carcinoma, breast cancer, osteosarcoma, and lately GBM, plays a functional role in glioma by sensitizing cells to radiation through impaired DNA repair, increased DNA double-strand breaks, G2/M arrest, and apoptosis, with its overexpression correlating with poor prognosis in patients receiving standard therapy (Liu et al., 2016). A recent study showed that TMZ treatment can increase the total and the phosphorylated form of c-Jun and c-Fos and eventually increase MMP9 to induce resistance to TMZ in GBM (Thanh et al., 2024). These findings give insight into why GBM becomes resistant to TMZ treatment over time and suggest that adding other agents to TMZ treatment may be beneficial for treatment success. Our results also showed that Fos was also increased in the treatment group, meaning that cells became resistant to TMZ over time.

In our heatmap, we observed that CDKN1A and CCN1 also possess prognostic value in GBM. Hu and colleagues reported that CDKN1A expression was correlated with clinicopathological parameters of GBM patients and has been implicated in AKT-mediated TMZ resistance in glioma cells (Hu et al., 2021). CCN1 is found to be involved in the enhanced invasiveness and MES phenotype transition of GSC, and in vitro and in vivo assays demonstrated that knockdown of CCN1 in MES-GSCs led to a reduction in tumor stemness, proliferation, and invasion (Guo et al., 2024). Moreover, CCN1 can enable apoptosis through TRAIL mediation or survivin downregulation in esophageal cancers (Dang et al., 2017, 2019). However, most of the studies conducted on GBM indicate that CCN1 and CDKN1A are targets as biomarkers and treatment should target these genes. Unexpectedly, our RNA-seq results showed that combination therapy increased these genes, but we think that these genes are increased in GBM cells to develop resistance to treatment and escape death. Therefore, this issue should be addressed in a broader molecular study.

Recent studies revealed that SOX10 downregulation can increase the proliferation and invasion of GBM cells and decrease the survival chance, so much so that even suppression of SOX10 induces a switch from the RTK I subtype to the mesenchymal (MES) subtype, which is associated with a worse prognosis for patients (Wu et al., 2020; Man et al., 2024). In our case, combinatorial treatment increased the SOX10 expression compared to the control GBM cells, meaning there is a change in the phenotype of GBM cells to a more favorable prognosis, but our findings need to be supported by future in vivo experiments to be reflected in the clinical setting. The use of the single cell line is a limitation of our work. To address this, future studies will aim to validate our findings using additional cell lines such as LN18 and LN229 as well as in vivo models.

The KEGG pathway defines “ribosome” as referring to “ribosome biogenesis,” a complex multistep process that initiates within the nucleolus and culminates in synthesizing functional ribosomes, which are indispensable for cellular proliferation (Elhamamsy et al., 2022; Wu et al., 2024). Clonogenic assay results demonstrated that TMZ-resistant cells retained the ability to form colonies. At the same time, the combination therapy exhibited the most pronounced effects on impairing cell proliferation and reducing the colony formation capacity of cancer cells. This finding was corroborated by RNA sequencing data, revealing that genes associated with ribosome biogenesis were more significantly upregulated in the control group compared to those subjected to the triple combination therapy.

In conclusion, in the present study we provide the first systematic evaluation of a triple combination therapy employing DNA-binding agents, notably epirubicin, to overcome TMZ resistance in GBM. The enhanced efficacy derived from low-dose combinations of TMZ, epirubicin, and 5-FU indicates a promising therapeutic strategy in the chemotherapy regimen for GBM. Although additional in vitro and in vivo investigations are essential to elucidate the underlying antitumor mechanisms, our findings highlight significant molecular alterations from combination therapy. This research lays the groundwork for repurposing these FDA-approved agents as a viable therapeutic approach for addressing drug-resistant GBM.

## Supplementary materials

Figure S1 Flow cytometry gating strategy and apoptosis analysis of U87 cells under different treatment conditions. Rows are labeled as follows: (A) Untreated, (B) TMZ 200 μM, (C) TMZ + 5-FU, (D) TMZ + epirubicin, and (E) TMZ + 5-FU + epirubicin. Columns show sequential gating steps: FSC vs. SSC to exclude debris, FSC-H vs. FSC-A to remove doublets, and annexin V (APC) vs. PI to identify viable (annexin V^−^/PI^−^), early apoptotic (annexin V^+^/PI^−^), late apoptotic (annexin V^+^/PI^+^), and necrotic (annexin V^−^/PI^+^) populations. Experiments were performed in biological triplicates; representative plots are shown.

Figure S2 The top 15 biological processes (BPs), molecular functions (MFs), and cellular components (CCs) are shown. Differentially expressed genes (DEGs) in TMZ + epirubicin + 5FU vs. control were primarily enriched in BPs related to the response to chemicals and MFs associated with aminoacyl-tRNA ligase activity. For CCs, DEGs were mainly enriched in the extracellular space, extracellular region, and amino acid transport complex (A). In TMZ + epirubicin + 5FU vs. TMZ, significantly enriched MF terms included structural molecule activity, structural component of the ribosome, and protein binding, while enriched CC terms were cytosolic ribosome, cytosolic large ribosomal subunit, and side of membrane (B).

**Table S:** Comparison groups performed and the numbers of genes that differ significantly.

Conrtol group	Experimental group	Upregulated group	Unchanging	Downregulated gene
**Control**	TMZ	1253	5510	1649
**Control**	Epirubicin	2535	3447	2430
**Control**	TMZ+ 5-FU	141	6711	168
**Control**	TMZ+ Epirubicin	864	4492	837
**Control**	TMZ+ Epirubicin+5-FU	2095	8103	1825
**TMZ**	TMZ+ 5-FU	1397	3214	1000
**TMZ**	TMZ+ Epirubicin	1939	6015	1718
**TMZ**	TMZ+ Epirubicin+ 5-FU	2655	4755	2543

## Data Availability

All data produced by this study can be provided upon reasonable request from the corresponding author.

## Footnotes

1 Alexa A, Rahnenfuhrer J (2024). topGO: Enrichment Analysis for Gene Ontology. R Package Version 2.58.0. https://bioconductor.org/packages/release/bioc/html/topGO.html [accessed 10.08.2025].
