# Supplementary figures and images for: Combinatorial effect of epirubicin and 5-fluorouracil in the treatment of temozolomide-resistant glioblastoma cells
Source: Turk J Biol. 2026 Mar 13;50(2):170–84. doi: 10.55730/1300-0152.2799 (PMC13124144; doi:10.55730/1300-0152.2799)

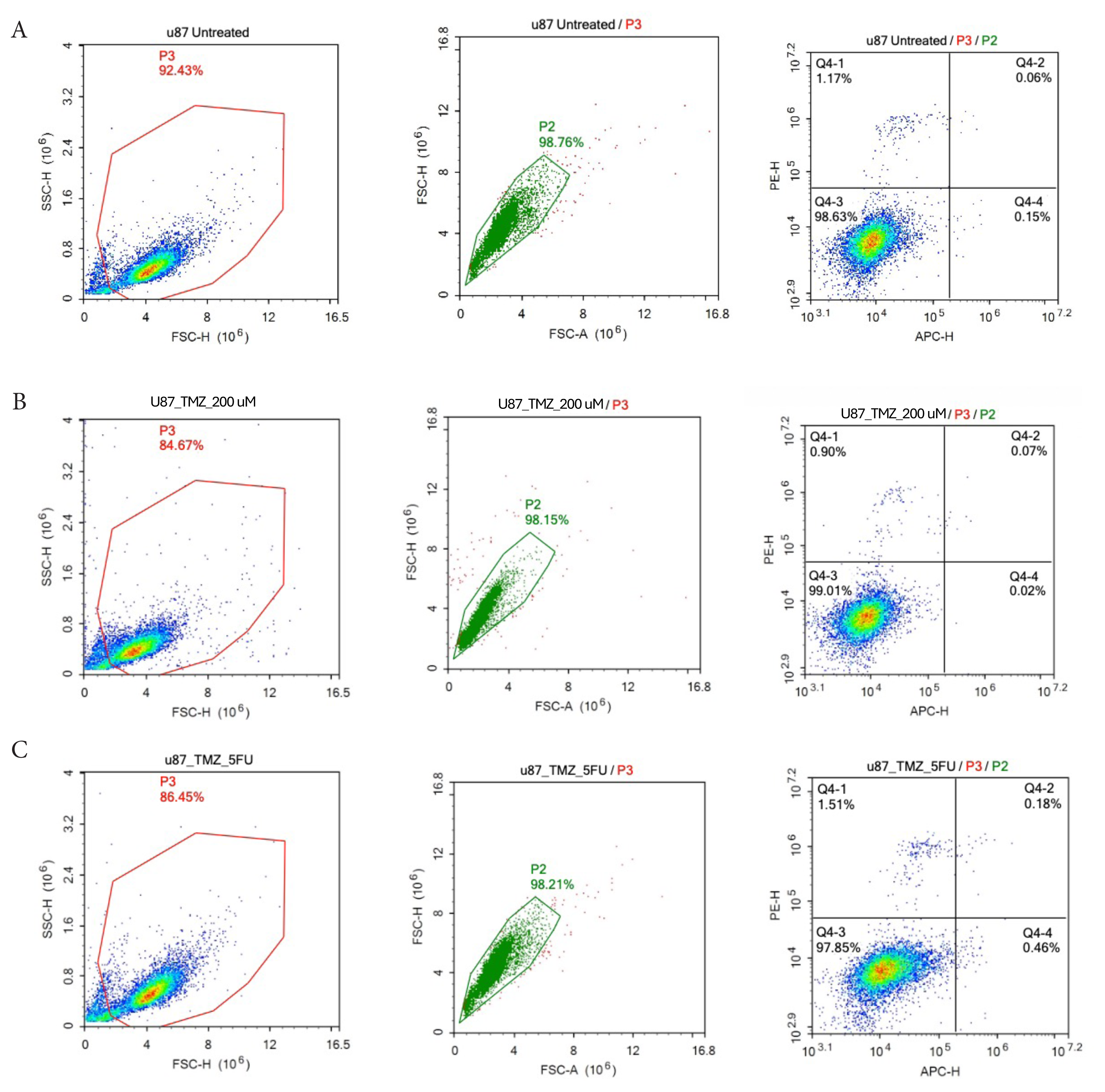

Supplement: Figure S1 — Flow cytometry gating strategy and apoptosis analysis of U87 cells under different treatment conditions. Rows are labeled as follows: (A) Untreated, (B) TMZ 200 μM, (C) TMZ + 5-FU, (D) TMZ + epirubicin, and (E) TMZ + 5-FU + epirubicin. Columns show sequential gating steps: FSC vs. SSC to exclude debris, FSC-H vs. FSC-A to remove doublets, and annexin V (APC) vs. PI to identify viable (annexin V−/PI−), early apoptotic (annexin V+/PI−), late apoptotic (annexin V+/PI+), and necrotic (annexin V−/PI+) populations. Experiments were performed in biological triplicates; representative plots are shown. [file tjb-50-02-170s1a.tif]

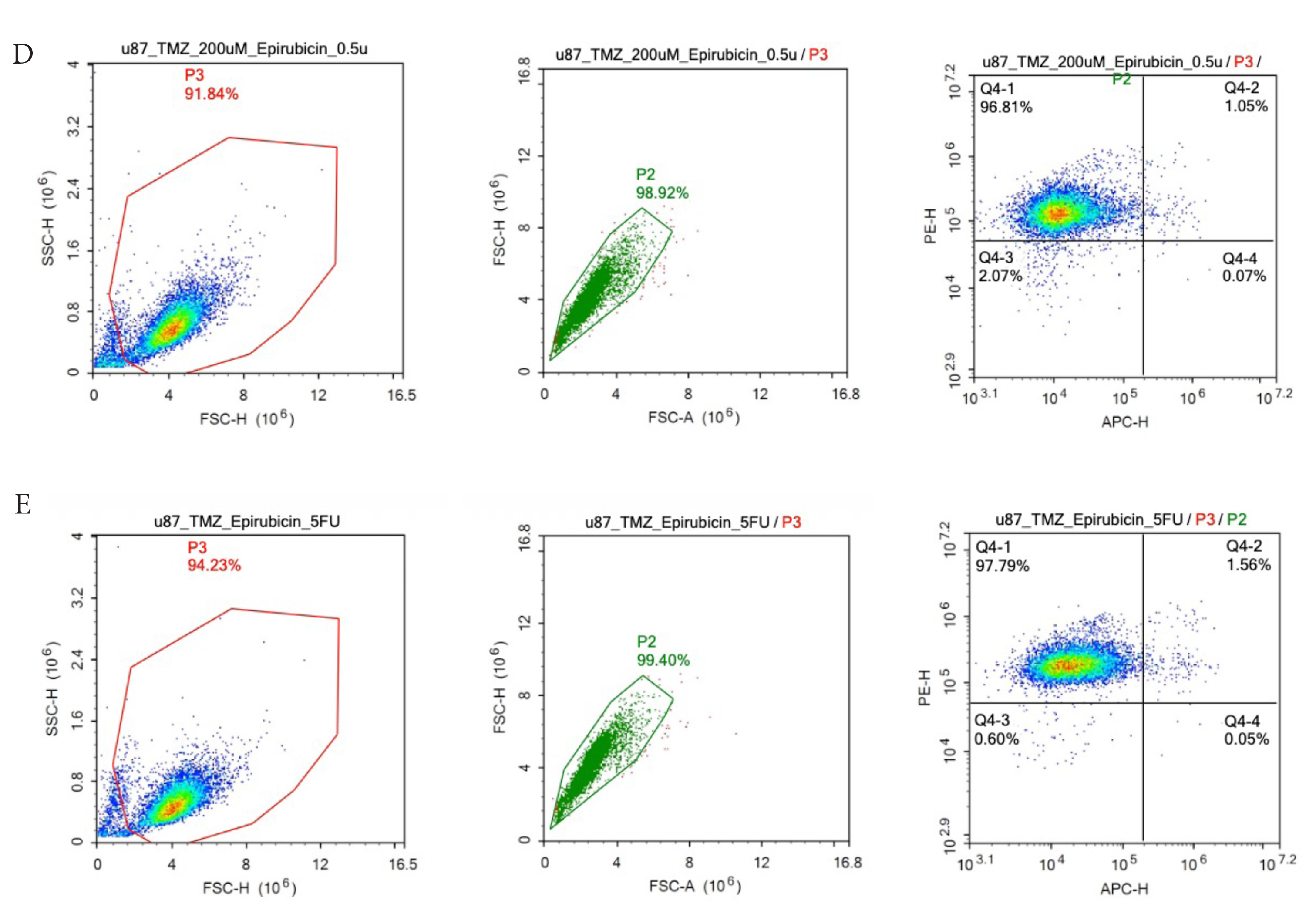

Supplement: Figure S1 — Flow cytometry gating strategy and apoptosis analysis of U87 cells under different treatment conditions. Rows are labeled as follows: (A) Untreated, (B) TMZ 200 μM, (C) TMZ + 5-FU, (D) TMZ + epirubicin, and (E) TMZ + 5-FU + epirubicin. Columns show sequential gating steps: FSC vs. SSC to exclude debris, FSC-H vs. FSC-A to remove doublets, and annexin V (APC) vs. PI to identify viable (annexin V−/PI−), early apoptotic (annexin V+/PI−), late apoptotic (annexin V+/PI+), and necrotic (annexin V−/PI+) populations. Experiments were performed in biological triplicates; representative plots are shown. [file tjb-50-02-170s1b.tif]

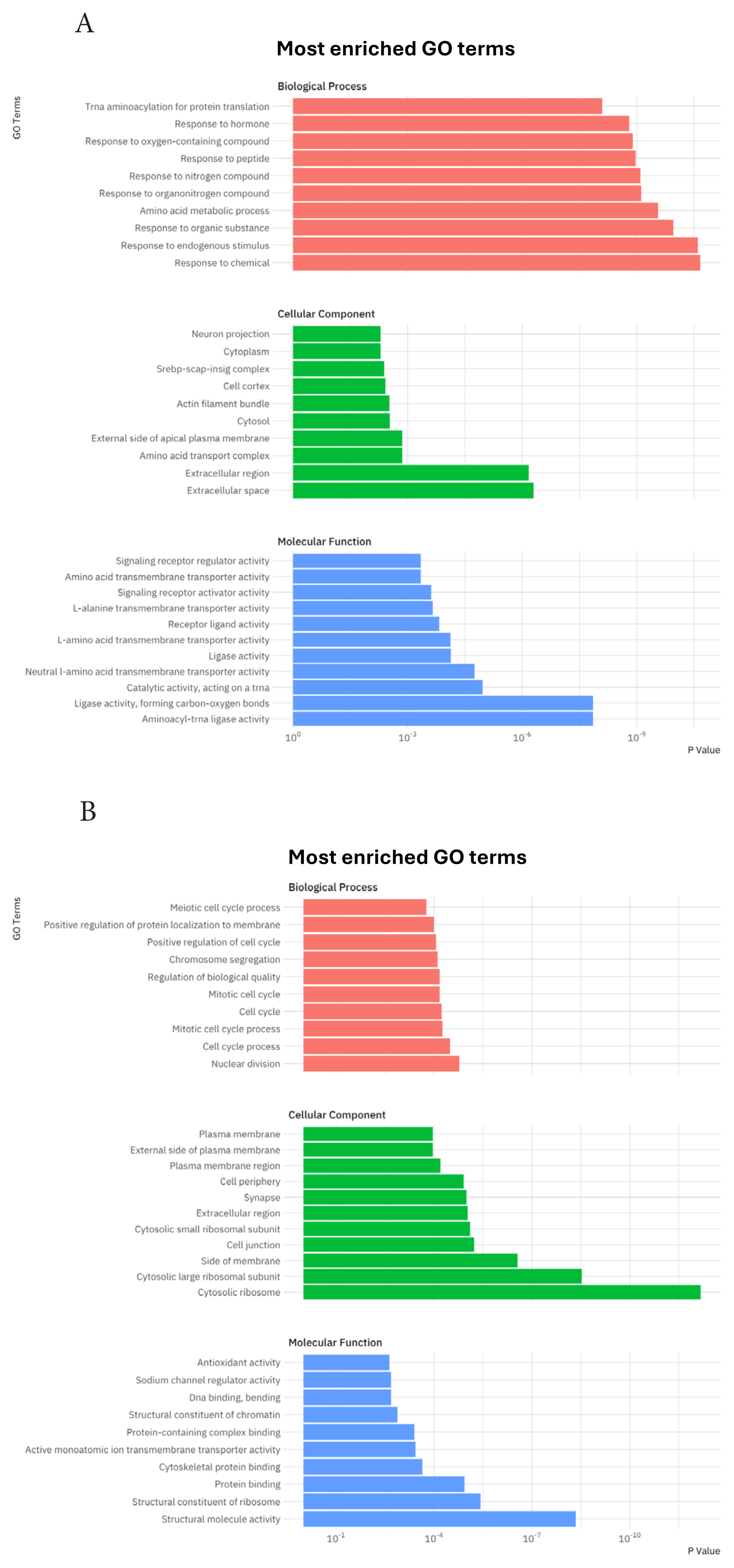

Supplement: Figure S2 — The top 15 biological processes (BPs), molecular functions (MFs), and cellular components (CCs) are shown. Differentially expressed genes (DEGs) in TMZ + epirubicin + 5FU vs. control were primarily enriched in BPs related to the response to chemicals and MFs associated with aminoacyl-tRNA ligase activity. For CCs, DEGs were mainly enriched in the extracellular space, extracellular region, and amino acid transport complex (A). In TMZ + epirubicin + 5FU vs. TMZ, significantly enriched MF terms included structural molecule activity, structural component of the ribosome, and protein binding, while enriched CC terms were cytosolic ribosome, cytosolic large ribosomal subunit, and side of membrane (B). [file tjb-50-02-170s2.tif]
